# The C-Terminal Region of G72 Increases d-Amino Acid Oxidase Activity

**DOI:** 10.3390/ijms15010029

**Published:** 2013-12-20

**Authors:** Sunny Li-Yun Chang, Chia-Hung Hsieh, Yen-Ju Chen, Chien-Ming Wang, Chung-Shiuan Shih, Pei-Wen Huang, Asif Mir, Hsien-Yuan Lane, Guochuan E. Tsai, Hao-Teng Chang

**Affiliations:** 1 Graduate Institute of Basic Medical Science, College of Medicine, China Medical University, No. 91, Hsueh-Shih Road, Taichung 40402, Taiwan; E-Mails: liyunchang@mail.cmu.edu.tw (S.L.-Y.C.); chhsiehcmu@mail.cmu.edu.tw (C.-H.H.); feeling00924@yahoo.com.tw (C.-M.W.); frog76420@gmail.com (P.-W.H.); 2 Department of Medical Laboratory Science and Biotechnology, China Medical University, Taichung 40402, Taiwan; E-Mails: campbiology@livemail.tw (Y.-J.C.); unicorn-star@hotmail.com (C.-S.S.); 3 Department of Bioinformatics & Biotechnology, Faculty of Basic and Applied Sciences, International Islamic University (IIU), H-10 Sector, Islamaba 44000, Pakistan; E-Mail: mir77uspk@hotmail.com; 4 Graduate Institute of Clinical Medical Science, China Medical University, Taichung 40402, Taiwan; E-Mail: hylane@gmail.com; 5 Department of Psychiatry, China Medical University Hospital, Taichung 40402, Taiwan; 6 Department of Psychiatry, Harbor-UCLA Medical Center and the Los Angeles Biomedical Research Institute, Torrance, CA 90502, USA; E-Mail: etsai@labiomed.org

**Keywords:** d-amino acid oxidase, G72, yeast-two hybrid

## Abstract

The schizophrenia-related protein G72 plays a unique role in the regulation of d-amino acid oxidase (DAO) in great apes. Several psychiatric diseases, including schizophrenia and bipolar disorder, are linked to overexpression of DAO and G72. Whether G72 plays a positive or negative regulatory role in DAO activity, however, has been controversial. Exploring the molecular basis of the relationship between G72 and DAO is thus important to understand how G72 regulates DAO activity. We performed yeast two-hybrid experiments and determined enzymatic activity to identify potential sites in G72 involved in binding DAO. Our results demonstrate that residues 123–153 and 138–153 in the long isoform of G72 bind to DAO and enhance its activity by 22% and 32%, respectively. A docking exercise indicated that these G72 peptides can interact with loops in DAO that abut the entrance of the tunnel that substrate and cofactor must traverse to reach the active site. We propose that a unique gating mechanism underlies the ability of G72 to increase the activity of DAO. Because upregulation of DAO activity decreases d-serine levels, which may lead to psychiatric abnormalities, our results suggest a molecular mechanism involving interaction between DAO and the *C*-terminal region of G72 that can regulate *N-*methyl-d-aspartate receptor-mediated neurotransmission.

## 1. Background

NMDA neurotransmission is the dominant molecular mechanism for synaptic plasticity, cognition, and memory. Many neurological and psychiatric diseases are associated with dysfunction of NMDA receptor-mediated neurotransmission (reviewed in [1]). Overexpression and hyperactivity of brain d-amino acid oxidase (DAO) have been associated with schizophrenia [2,3]. Hyperfunction of DAO, resulting in a decrease in the d-serine level and hypofunction of the NMDA receptor, has been proposed to be an underlying dysregulation of the disease [4,5]. In serum [6] and cerebrospinal fluid [7] of patients with schizophrenia, d-serine levels are reduced. In addition to its role in psychosis and cognition, DAO may be involved in amyotrophic lateral sclerosis, a neuronal disease involving the progressive loss of motoneurons; specifically, a single nucleotide polymorphism that yielded the dysfunctional point mutation R199→W was found in the gene encoding DAO in the genomes of a family with the disease [8], resulting in an accumulation of d-serine, excitotoxicity, and motoneuron death. Manipulation of DAO activity could be an approach to regulate d-serine levels [9]. Given the importance of DAO regulation in CNS disorders, studies of the molecular mechanism underlying the endogenous DAO activity regulation could provide potential therapeutic benefits. For example, DAO inhibitors may provide a valuable therapeutic strategy to improve schizophrenia symptoms due to low NMDA function (reviewed in [10]).

Recently, a primate-specific protein G72 has been characterized as a modulator of DAO activity [11]. The genetic variations of G72 were reported to contribute to various CNS diseases associated with glutamatergic signaling dysfunction [11–13]. Expression of G72 gene in transgenic mice induced behavioral phenotypes that are related to schizophrenia [14,15]. The gene encoding G72, like that for DAO, is a susceptibility gene for schizophrenia and bipolar disorder [11,16,17]. The missense single nucleotide polymorphism rs2391191 in G72, encoding a G→A mutation, is a possible genetic feature of schizophrenia [11,18–20]. Genetic association studies on major depression and bipolar disorder also suggested rs2391191 to be a risk polymorphism [21]. Furthermore, a relatively high G72 level in serum may be a molecular feature for schizophrenia [22].

Although the important role of G72 on DAO regulation has been suggested [11], the specific role G72 plays in regulating DAO activity in patients with schizophrenia is controversial. Biochemical studies by Sacchi and colleagues revealed that a 1:2 molar ratio of G72 to DAO is optimal for DAO inhibition [23]. Conversely, Chumakov *et al.* demonstrated that G72 enhances DAO activity, resulting in a reduction in the concentration of d-serine [11]. We have discovered that G72 is elevated in the serum of medicated or non-medicated schizophrenia patients [20], despite the observation that G72 mRNA and G72 are hardly detectable in the brain [23,24]. Consequently, NMDA dysfunction and its related pathological symptoms of schizophrenia may result from overexpression of G72 that changes DAO activity. To characterize the possible roles of G72 and DAO, the molecular interaction between G72 and DAO and the underlying molecular mechanism involving G72 regulation of DAO need to be studied. In this study, we identified the regions within G72 that impact on DAO activity. We also explored the interaction between G72 and DAO by a molecular docking exercise.

## 2. Results

**Figure 1.**
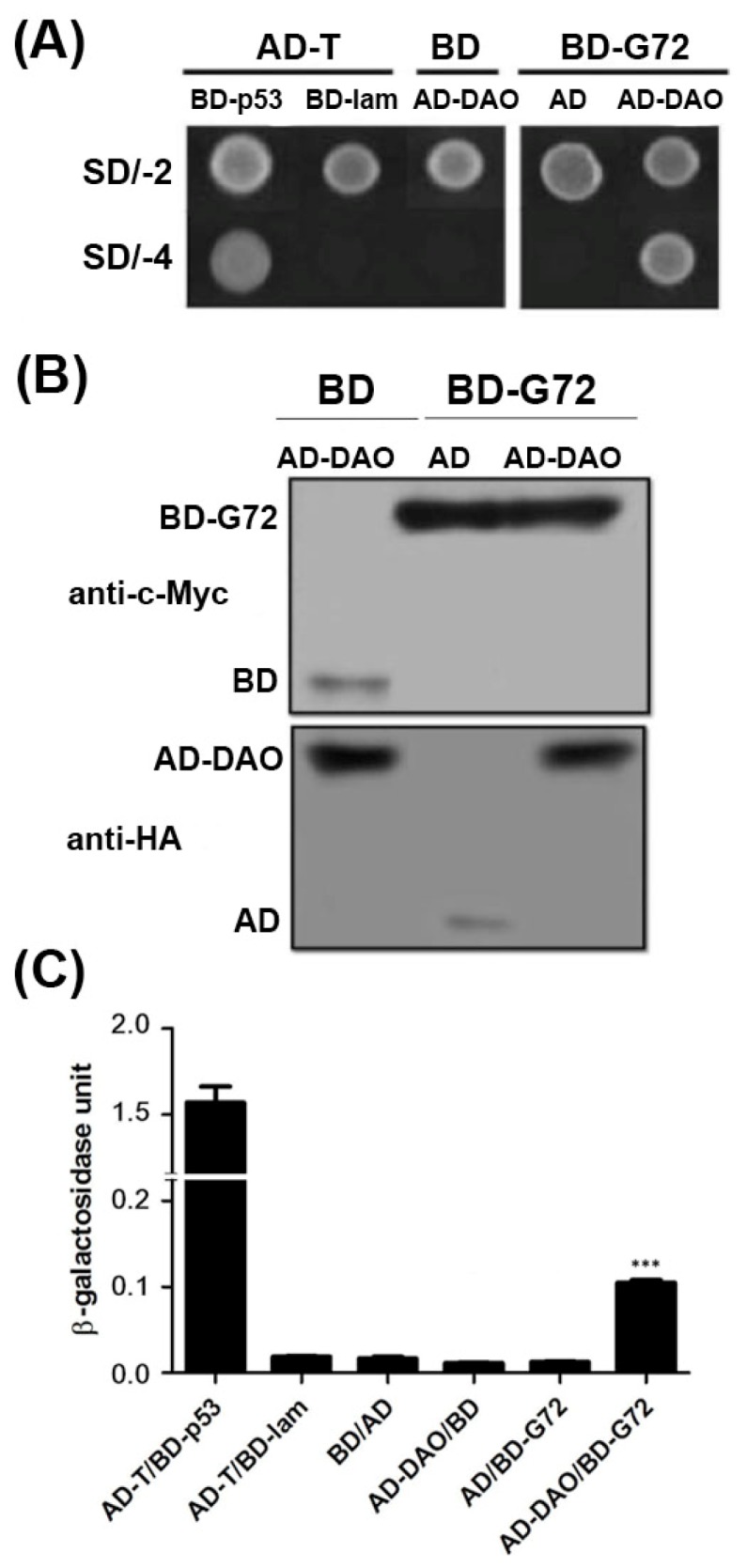
G72 interacts with d-amino acid oxidase (DAO). (**A**) Yeast co-transformed with plasmids containing the genes for AD-DAO and BD-G72 were viable on SD/-2 and SD/-4 plates; (**B**) Western blotting confirmed that AD-DAO and BD-G72 were expressed in the yeast system. Anti-c-Myc was used to detect the DNA-binding domain (BD), and anti-HA was used to detect the activation domain (AD); and (**C**) Relative interaction strength between DAO and G72 was determined using the β-galactosidase activity assay. *** *p* < 0.001 compared with the interaction of BD and AD.

G72 and DAO can interact as demonstrated by colocalization studies in mammalian cells, yeast-two hybrid studies in yeast cells and BiaCore experiments *in vitro* [11]. To confirm the interaction between G72 and DAO, we showed that transformants containing the AD-DAO and BD-G72 plasmids were viable on SD/-4 plates, which indicated that AD-DAO and BD-G72 interacted with each other (Figure 1A, top panel). Control experiments showed that AD-DAO and BD-G72 did not interact with BD and AD tags in yeast, respectively. These positive and negative controls confirmed that the yeast two-hybrid experiments are functioning as expected as the findings in AD-T/BD-p53 and AD-T/BD-lam showed respectively. A reason that transformants, in general, are not viable on SD/-4 plates is because the recombinant proteins are not expressed in yeast cells. We used Western blotting to confirm that the fusion proteins were expressed in yeast. The BD tag and BD-G72 were detected by anti-c-Myc (Figure 1B, upper panel), and the AD tag and AD-DAO were detected by anti-HA (Figure 1B, lower panel). Furthermore, the relative strengths of the interactions between the various constructs containing an AD or BD tag were quantified by the β-galactosidase assay. If the relative strength of the AD and BD tag interaction as 1 fold, the AD-DAO/BD-G72 interaction was 5.7-fold stronger (Figure 1C). Taken together, the experiments indicated that AD-DAO and BD-G72 were expressed and interacted in the yeast cells and that the interaction could be monitored using the SD/-4 plate and β-galactosidase activity assays.

### 2.1. The *C*-Terminal Region of G72 Is Essential for the Interaction between G72 and DAO

**Figure 2.**
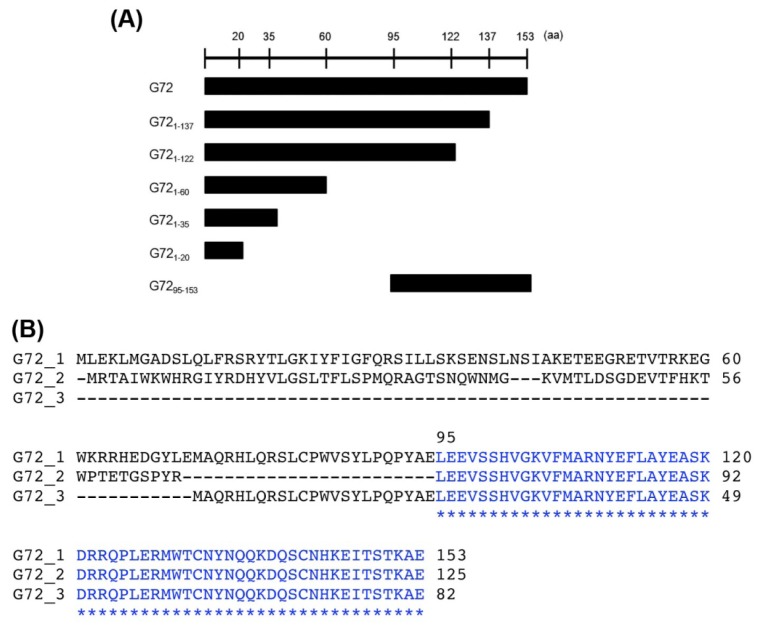
Truncated fragments of G72. (**A**) Schematics of the G72 constructs co-transfected with activation domain (AD)-DAO in yeast; and (**B**) The amino acid sequence alignment of the three G72 isoforms. The residues of the *C*-terminal sequence that are identical in the isoforms are shown in blue.

**Figure 3.**
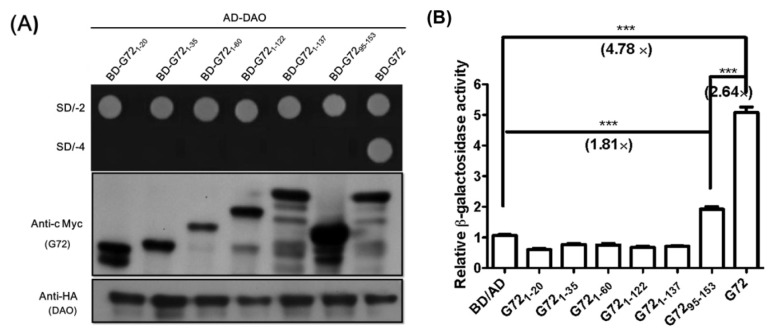
The *C*-terminal region of G72 interacts with DAO. (**A**) Upper panel. Only yeast co-transformed with plasmids containing the genes for AD-DAO and BD-G72 were viable on SD/-2 and SD/-4 plates. Yeast co-transformed with AD-DAO and a truncated form of the BD-G72 gene was not viable on SD/-4 plates. Lower panel. Western blotting to confirm that AD-DAO and BD-G72 and truncated forms of BD-G72 were expressed in the yeast system; and (**B**) The relative strengths of the interactions between DAO and G72 or a truncated form of G72 were determined by the level of β-galactosidase activity and normalized to the activity in yeast co-transformed with the AD and BD tags. *** *p* < 0.001.

Three isoforms of G72 (isoform 1: GI:126362975, isoform 2: GI:240120173, isoform 3: GI:240120029) are found in the Protein database in NCBI (http://www.ncbi.nlm.nih.gov/protein/). The longest G72, isoform 1, contains 153 amino acid residues, but no known functional motifs or domains have been identified. A conserved *C*-terminal region is found in all G72 isoforms, which suggests that the region is important for G72 function and/or folding. We therefore characterized the effects of *C*-terminally truncated fragments of G72 on DAO activity. The truncated fragments, BD-G72_1–20_, BD-G72_1–35_, BD-G72_1–60_, BD-G72_1–122_, BD-G72_1–137_, and BD-G72_95–53_ (Figure 2A), were constructed and co-transformed with AD-DAO into AH109 yeast cells. The first five truncated segments were designed so as not to destroy the individual G72 secondary structures, which had been predicted by HNN Secondary Structure Prediction Method at http://npsa-pbil.ibcp.fr/cgi-bin/npsa_automat.pl?page=/NPSA/npsa_hnn.html (Figure S1). G72_95–153_ contains the conserved sequence found in the three G72 isoforms (Figure 2B). Interestingly, only transformants containing full-length BD-G72, in addition to AD-DAO, were viable on SD/-4 plates (Figure 3A, upper panel) even though AD-DAO and the BD-G72 truncated variants were expressed in AH109 cells (Figure 3A, middle and lower panels). Therefore, residues 1–137 of the longest isoform of G72 may not interact with DAO and, instead, residues found in G72_138–153_ may interact with DAO.

Interactions involving short peptides similar to G72_138–153_ are difficult to discern using the yeast two-hybrid technique. Although the transformant containing AD-DAO and BD-G72_95–153_ did not grow on SD/-4 plates (Figure 3A, upper panel), the β-galactosidase activity for this transformant was 1.81-fold that of the baseline BD/AD tag activity (Figure 3B) and 38% that of the activity found for the AD-DAO/BD-G72 system, indicating that the *C*-terminal region of G72 may interact with DAO and that the region(s) encompasses residues 123 and 153 and/or 138 and 153.

### 2.2. G72_123–153_ and G72_138–153_ Interact with DAO

**Figure 4.**
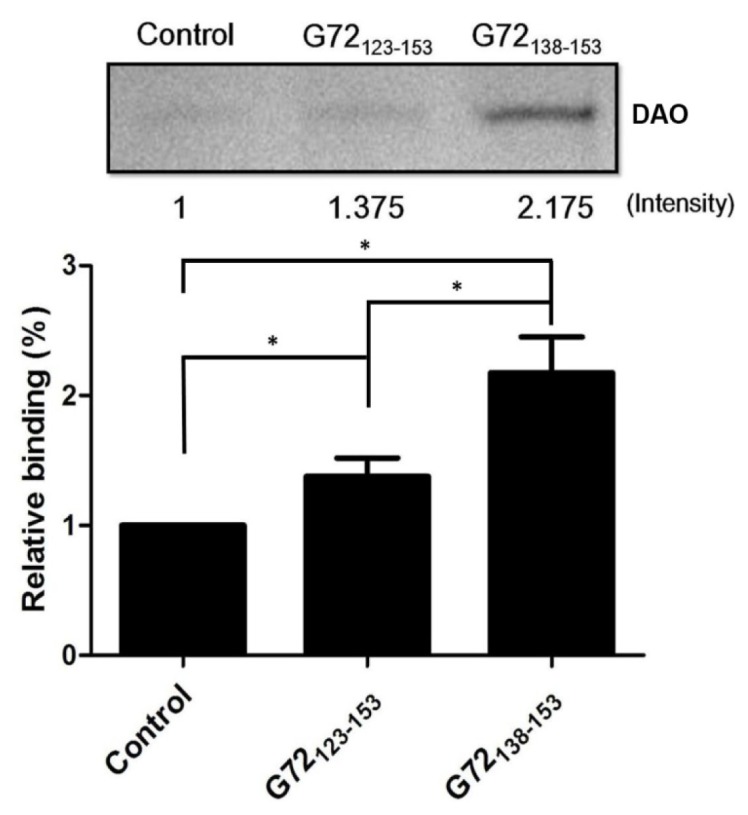
Pull-down assay to assess the interactions between DAO and G72_123–153_ or G72_138–153_. DAO was mixed with biotinylated G72_123–153_ or G72_138–153_. After isolating the pulled-down fraction, the amount of recovered DAO was determined by SDS-PAGE with the relative binding ability of each peptide determined by the intensity of the corresponding DAO band. * *p* < 0.05.

To confirm that G72 residues between 123 and 153 are involved in DAO binding, *N*-terminally biotinylated G72_123–153_ and G72_138–153_ were synthesized for use in an *in vitro* streptavidin pull-down assay. After incubation with streptavidin beads, recombinant DAO, and one of the biotinylated peptides, the beads were isolated by centrifugation and washed with PBS to isolate the bound proteins, which were subjected to SDS-PAGE (Figure 4). A very minor band was observed when DAO was incubated with only the beads. The relative amounts of DAO pulled down by G72_123–153_ or G72_138–153_ were 1.375-fold and 2.175-fold greater than that found for the control, respectively. Residues between 138 and 153 are, therefore, likely to be involved in the DAO/G72 interaction, which regulates DAO activity.

### 2.3. DAO Activity Is Increased by G72 Truncation Mutants

**Figure 5.**
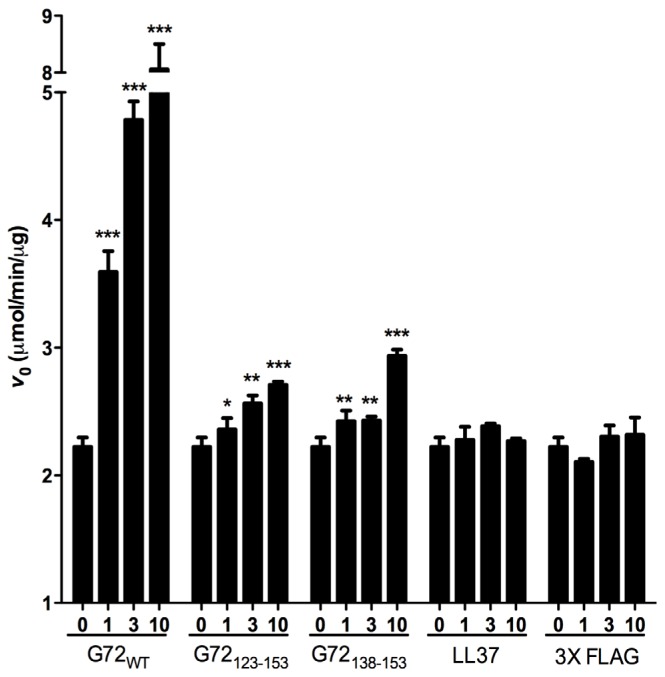
G72, G72_123–153_, and G72_138–153_ enhance DAO activity. The initial velocity (*V*_0_) of DAO activity was enhanced by G72, G72_123–153_, and G72_138–153_ but not by the control peptides LL37 and 3× FLAG. *** *p* < 0.001; ** *p* < 0.01; * *p* < 0.05.

According to the results of the yeast two-hybrid and pull-down assays, DAO interacts with G72_123–153_ and G72_138–153_. We also assessed the effects of the G72 peptides on DAO activity. Because it has been suggested that G72 regulates DAO activity [11,23], we assessed how G72_123–153_ and G72_138–153_ to affect the *V*_0_ of DAO activity. For the enzymatic assay, two unrelated peptides LL37 and 3× FLAG served as negative controls. LL37 is a 37-residue positively charged peptide, and 3× FLAG is a 23-residue negatively charged peptide. Compared with *V*_0_ when DAO was present alone or with one of the control peptides, full-length G72 and both *C*-terminal G72 peptides increased *V*_0_ in a dose-dependent manner (Figure 5). At 10 μM G72, G72_123–153_, or G72_138–153_, *V*_0_ increased by 355%, 22% or 32%, respectively (Table 1). Taken together the β-galactosidase and pull-down assay results, residues between 138 and 153 of G72 appear to be involved in enhancement of DAO activity. However, this region of G72 alone is not sufficient to account for the activity induced by full-length G72.

### 2.4. Docking of DAO with G72 Peptides

**Figure 6.**
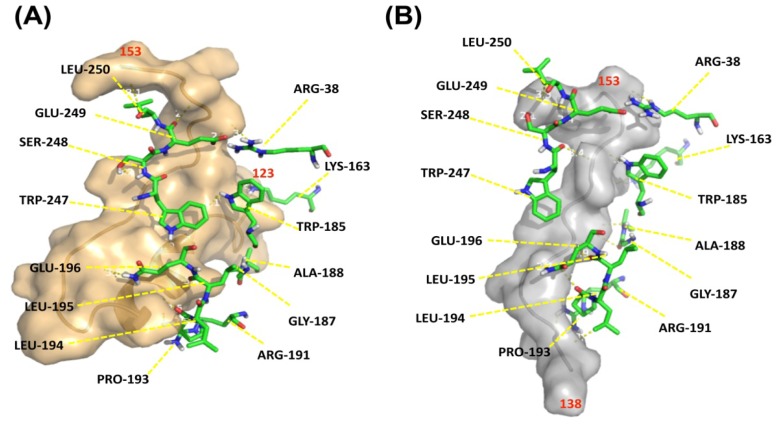
G72_123–153_ and G72_138–153_ bind at similar sites on DAO. The predicted structures of (**A**) G72_123–153_ (brown ribbon diagram) and (**B**) G72_138–153_ (grey ribbon diagram) were docked with DAO (the DAO residues are shown as stick models). DAO residues that reside within 3.5 Å of residues in the G72 peptides are labeled (see Results for the involved DAO residues).

To investigate how G72 and DAO interact, an *in silico* docking approach was employed. Because the structure of G72 is unavailable, the three-dimensional structure of G72 was predicted [25] by MODELLER 9v10, a comparative modeling technique [26]. Residues 123–153 and 138–153 were extracted from the predicted structure and individually docked to DAO. The fragment of residues 123–153 consisted of loop-helix-loop conformation (Figure 6A, brown ribbon diagram) and loop conformation was predicted in residues 138–153 (Figure 6A, grey ribbon diagram). Residues Arg38, Lys163, Trp185, Gly187, Ala188, Arg191, Pro193–Glu196, and Trp247–Leu250 in DAO, which are loop residues, interact with both of the docked G72 peptides (Figure 6, backbones).

## 3. Discussion

Despite that the importance of DAO regulation in neurophysiologic functions has being revealed, the underlying mechanism remains unclear. Molecular characterization of G72, the newly identified endogenous modulator of DAO activity, may provide a better understating of the regulation of DAO activity. We have performed studies in determining the DAO-interactive domains of G72. Using yeast-two hybrid analysis, we demonstrated that G72 could interact with DAO via its *C*-terminal region, but not the *N*-terminus. We further identified two short motifs, G72_38–153_ and G72_123–158_, which can interact with DAO and enhance the DAO activity as evidenced by the pull-down assay and *in vitro* DAO activity assay, respectively. Our truncation study indicates that residues within the *C*-terminal region are essential for DAO regulation, and the *N*-terminal G72 constructs did not interact with DAO. Nevertheless, the G72 peptides containing residues 123/138 to 153 did not interact with DAO as strongly as full-length G72 did, in terms of enhancing DAO activity; therefore, other regions of G72 are likely involved in regulating DAO, too.

According to the three-dimensional structure of DAO, four loops are found at the entrance of the tunnel that is part of the substrate and cofactor active site. G72_38–153_ and G72_123–158_ interact with three of the four loops according to our docking exercise. Such an interaction can cause a conformational change that enhances the rate of substrate entry and/or release of products. The docked model, involving G72_123–153_, contains loop**-**helix-loop conformation and maintains a hairpin shape. Theoretically, the structure of G72_123–153_ might be less flexible than that of G72_138–153_, which forms an entire long loop. However, loop regions often serve as the binding sites in protein-protein interactions [27]. Therefore, the G72_138–153_ peptide may have sufficient flexibility to accommodate DAO and thereby enhance the enzymatic activity of DAO. Similarly, the antimicrobial peptide LL37, with 37 residues, interacts with Xog1p, a β-1,3-exoglucanase, thereby enhancing the exoglucanase activity, destruction of fugal cell walls by Xog1p and consequently inhibiting fungal adhesion on plastic [28].

Detection of endogenous G72 expression in human brain tissue has been reported by Sacchi and colleagues [23]. Native G72 protein was detected in pellet fraction of human brain biopsies by Western blotting. Notably, they mentioned that the expression level of native G72 is very low and a large amount of human brain tissues were required to confirm the G72 expression [23]. This low level of expression may explain why no successful *in situ* detection of endogenous G72 mRNA and protein expression has been reported so far. Another possibility could also be due to the technical limitation, *i.e.*, lacking the high affinity antibody against G72. In addition, to analyze the potential interaction of G72 and DAO *in vivo*, Sacchi and colleagues demonstrated the exogenous G72 and DAO colocalize in the external membrane of mitochondria in glial cells by using confocal microscopy observation and Fluorescence Resonance Energy Transfer-based assay [29]. However, they reported that G72 inhibits DAO activity *in vitro* [23] which is opposite to our findings and Chumakov’s report of enhancing activity [11]. Our study shows that either full-length G72 or the two short *C*-terminal G72 peptides could activate DAO. The difference may be due to the temperatures during enzymatic reaction. Chumakov’s and our group performed the *in vitro* enzymatic reaction at 25 °C, a more relevant temperature for oxidative reaction than 4 °C chosen in Sacchi’s experiment. Nevertheless, the regulatory roles of G72 on DAO activity have not been fully explored; the functional relationship between G72 and DAO needs to be further investigated.

It has been suggested that manipulation of DAO activity can be an option of treatment for CNS disorders [30]. Our informatics docking results provide fundamental cues for designing synthetic peptides or chemical compounds to interrupt the interaction of G72 and DAO and to reduce the DAO activation since G72 enhance of DAO activity, as demonstrated by our studies. Based on the residues identified in the interaction between G72 and DAO, the negative regulators of DAO activity can be designed by docking *in silico* first, which can occupy the G72-interacting interfaces on DAO, before they are confirmed by *in vitro* and *in vivo* studies. d-serine plays a critical role in glutamate NMDA neurotransmission and its level must be maintained within a relatively narrow range to avoid under- or over-activating NMDA function, both of which can impair CNS function [31]. In schizophrenia, the serum level of d-serine is 17.7% lower than normal controls, and the ratio of serum d-serine to total serine is 28.4% lower [6]. This “subtle” dysregulation of d-serine can be “corrected” by the enhancement of DAO activity like G72_138–153_.

## 4. Methods

### 4.1. Constructs

DNAs encoding full-length DAO (1044 bp, GI:30524341), full-length G72 (462 bp, GI:126362975), and the G72 fragments G72_1–137_ (411 bp) and G72_95–153_ (282 bp) were PCR-amplified using the primer sets: DAO-F′-*Nde*I/DAO-R′-*Xho*I, G72-F′-*Nde*I/G72-R′-*Pst*I, G72-F′-*Nde*I/G72_1–137_-R′-*Pst*I, and G72_95–153_-F′-*Nde*I/G72-R′-*Pst*I, respectively (Table 2). The DAO and G72 cDNAs were individually cloned into pGADT7 or pGBDT7 (Clontech, Mountain View, CA, USA) so that the expressed DAO and G72 would be *N*-terminally tagged with the activation domain (AD) or binding domain (BD), respectively. To generate pGBDT7 plasmids containing the genes for the BD-tagged, G72-truncated variants, the following primer sets were used: for G72_1–20_ (60 bp), G72-60-F′/G72-60-R′; for G72_1–35_ (105 bp), G72-105-F′/G72-105-R′; for G72_1–60_ (180 bp), G72-180-F′/G72-180-R′; and for G72_1–122_ (366 bp), G72-366-F′/G72-366-R′.

To express the recombinant proteins, the DAO and G72 cDNAs were amplified using the primer sets DAO-F′-*Nde*I/DAO-R′-*Xho*I and G72-F′-*Nde*I/G72-R′-*Xho*I, respectively. The amplified PCR products were each digested with *Nde*I and *Xho*I and then inserted into pET23a(+) individually (Novagen, Darmstadt, Germany) so that the *C*-termini of the recombinant proteins were tagged with (His)_6_.

### 4.2. Yeast Two-Hybrid and β-Galactosidase Assays

The recombinant plasmid encoding AD-DAO was co-transformed with that for BD-G72 or with one of the plasmids encoding a truncated BD-G72 into *Saccharomyces cerevisiae* AH109 competent cells (Clontech, Mountain View, CA, USA) using Frozen-EZ Yeast Transformation II kit reagents (Zymo Research, Irvine, CA, USA). Each transformant (200 μL) was spread onto SD/-Leu/-Trp (SD/-2) and SD/-Leu/-Trp/-Ade/-His (SD/-4) plates, and the plates were incubated at 30 °C for 3 to 4 days. AD-T/BD-p53 and AD-T/BD-lam co-transformants served as the positive and negative controls, respectively.

β-Galactosidase activity was the measure of the interaction between AD-DAO and the BD-G72 constructs. Each type of AH109 transformant was individually cultured in SD/-2 broth at 30 °C with shaking at 230 rpm until the value of the culture *OD*_600_ approached 0.8. The exact *OD*_600_ value was recorded before the cells were harvested. The cells from a culture were transferred into three 1.5-mL microcentrifugation tubes and then centrifuged. The supernatants were carefully removed, and the cells were washed twice with 1 mL of Z buffer (16 mM Na_2_HPO_4_, 40 mM NaH_2_PO_4_, 10 mM KCl, 1 mM MgSO_4_, pH 7.0). The pellets were suspended in 0.3 mL of Z buffer. Then, 100 μL of each cell suspension was transferred into a new 1.5-mL tube and subjected to three cycles of freeze-thaw (liquid nitrogen and 37 °C water bath). The control contained 0.1 mL of Z buffer. Each sample was mixed with Z buffer (700 μL) containing 0.027% (*v*/*v*) β-mercaptoethanol. *p*-Nitrophenyl-α-d-galactopyranoside (160 μL, 4 mg/mL, Sigma, Steinheim, Germany) dissolved in Z buffer was added to each sample. The *OD*_450_ value was immediately determined and served as the blank. The samples were incubated at 30 °C until a yellow color developed. The reactions were stopped by adding 0.4 mL of 1 M Na_2_CO_3_, and the elapsed time was recorded. After centrifugation at 25 °C, the *OD*_450_ values of the supernatants were recorded. Measurement of β-galactosidase activity was defined according to the manufacturer’s parameters (Clontech, Mountain View, CA, USA).

### 4.3. Western Blotting

Expression of the AD-DAO and BD-G72 fusion proteins in yeast was confirmed by Western blotting. Each transformed yeast lysate was extracted in 8 M urea, 5% (*w*/*v*) SDS, 40 mM Tris-HCl, pH 6.8, 0.1 mM EDTA, 0.4 mg/mL bromophenol blue, 1% (*v*/*v*) β-mercaptoethanol, 1 mM proteinase inhibitor cocktail (Amresco, Solon, OH, USA), 20 mM phenanthroline, 0.5 mM benzamidine, and 1 mM phenylmethylsulfonyl fluoride with glass bead vortexing. After vortexing and heating at 95 °C, the supernatant was isolated by microcentrifugation, subjected to sodium dodecyl sulfate-polyacrylamide gel electrophoresis (SDS-PAGE; 15% *w*/*v* acrylamide), and transferred onto a polyvinylidene difluoride membrane (Pall, Pensacola, FL, USA). The membrane was blocked using 3% (*w*/*v*) bovine serum albumin dissolved in 137 mM NaCl, 2.7 mM KCl, 10.2 mM Na_2_HPO_4_, 1.8 mM KH_2_PO_4_, 0.1% (*w*/*v*) Tween-20, pH 7.4 for 1 h and then probed with mouse monoclonal antibodies against c-Myc (1:1000, Santa Cruz Biotechnology, Dallas, TX, USA) and HA (1:1000, Santa Cruz Biotechnology) tags overnight. To detect DAO-6H in a pull-down assay, monoclonal anti-His-tag (1:1000, Santa Cruz Biotechnology) was used instead. After washing three times with the same solution, the primary antibodies were probed with horseradish peroxidase-conjugated rabbit anti-mouse antibodies (1:5000, Jackson ImmunoResearch, West Grove, PA, USA) for 1 h, and then the membrane was washed with the same buffer three times. After adding enhanced chemiluminescence reagent (Millipore Corporation, Billerica, MA, USA), luminescent signals were detected and captured using a luminescent charge-coupled camera (Fujifilm, Tokyo, Japan).

### 4.4. Recombinant Protein Expression and Purification

pET23a-DAO and pET23a-G72 were transformed into *Escherichia coli* BL21(DE3)pLysS cells separately (Novagen, San Diego, CA, USA), which were screened on Luria-Bertani plates containing 100 μg/mL carbenicillin (MBio, New Taipei City, Taiwan) and 50 μg/mL chloramphenicol (MBio) and then cultured in the same medium at 37 °C overnight. When the *OD*_600_ value of each culture approached 0.8, isopropyl β-d-1-thiogalactopyranoside (1 mM for DAO-6H and 0.5 mM for G72-6H) was added. For DAO-6H expression, the *E. coli* were incubated at 25 °C for 16 h and then suspended in 20 mM HEPES, pH 7.9, 500 mM NaCl, 1 mM benzamidine, 1 mM phenylmethylsulfonyl fluoride. The cell suspension was sonicated, and the soluble fraction was isolated by centrifugation (12,000× *g*, 4 °C, 30 min). Recombinant DAO was purified using an ÄKTA Prime system with a HisTrap FF column (GE Health, Uppsala, Sweden).

G72-6H formed inclusion bodies. G72-6H was expressed at 37 °C for 4 h before harvesting the cells and isolated in inclusion bodies by homogenization and microcentrifugation. The pellets were harvested for further protein refolding procedure. To refold G72-6H, the inclusion bodies were dissolved in 0.5% (*w*/*v*) *N*-lauroylsarcosine, 1 mM dithiothreitol, 50 mM CAPS, pH 11.0, and 1 mM β-mercaptoethanol at 25 °C. After shaking for 1 h, the sample was dialyzed against 100 mM NaCl, 20 mM HEPES, pH 8.0, 1 mM β-mercaptoethanol containing 0.1%, then 0.05%, and finally, 0.005% (*w*/*v*) *N*-lauroylsarcosine sequentially at 4 °C [32]. Each step was for at least 6 h. Refolded G72 was stored at −20 °C.

### 4.5. Pull-Down Assay

Streptavidin Sepharose beads (20 μL, GE Healthcare, Uppsala, Sweden) were mixed with 10 μg of synthesized, biotinylated G72_123–153_ or G72_138–153_. Beads alone without incubation with synthetic peptides were used as negative control. Subsequently, 10 μg of recombinant DAO-6H in phosphate-buffered saline was added into each mixture so that its final volume was 500 μL. The mixtures were incubated at 4 °C for 3 h and then centrifuged at 3000× *g* for 1 min. The pellets were each washed ten times with 1 mL phosphate-buffered saline, then mixed with 20 μL of 80 mM Tris-HCl (pH 6.8), 2% (*w*/*v*) SDS, 10% (*v*/*v*) glycerol, 0.05% (*v*/*v*) β-mercaptoethanol and 0.2 mg/mL bromophenol blue and boiled. The samples were then subjected to 10% SDS-PAGE followed by Western blotting.

### 4.6. DAO Activity Assay

Recombinant DAO-6H (400 nM) was added into 3% (*w*/*v*) *o*-phenylenediamine, 1 U horseradish peroxidase, 40 mM d-alanine (final volume, 200 μL). The reaction at 25 °C generated hydrogen peroxide that was oxidized by the peroxidase and further converted, in the presence of *o*-phenylenediamine, to 2,3-diaminophenazine, which was quantified by measuring *OD*_450_. For initial velocity (*V*_0_) assays, 0, 1, 3, or 10 μM of G72_123–153_, G72_138–153_, LL37, or 3× FLAG was also present in the reaction buffer. LL-37 and 3× FLAG served as negative controls. Immediately after adding DAO-6H into each reaction, its *OD*_450_ was recorded for 1 min. One *V*_0_ unit was defined as the production of one micro mole hydrogen peroxide per minute per micro gram DAO.

### 4.7. Docking of DAO and G72 Peptides

Because a three-dimensional structure for G72 is unavailable, we employed a predicted structure of G72 modeled previously [25] for the docking analysis. DAO structural information was retrieved from the Protein Data Bank (PDBID 2E48) and used as ClusPro [33,34] input with residues 123–153 or 138–153 extracted from the modeled G72 structure. Atoms of DAO and each of the peptides with a distance separation <3.5 Å were defined as interacting residues.

## 5. Conclusions

Manipulation of the regulatory mechanisms of DAO activity is a novel approach to modulate NMDA function which is dysregulated in CNS disorders. We report G72 can interact with DAO and consequently enhance the DAO activity. We also demonstrate that the *C*-terminus of G72 is essential for this activation. Our results provide an understanding of the regulatory role of G72 in DAO activity.

## Supplementary Information



## Figures and Tables

**Table 1.** Initial velocity (*V*_0_) of DAO in the presence of G72-related or non-related peptides.

Peptide (M)	*V*_0_ of DAO (mol hydrogen peroxide produced/min/g DAO)			

	0	1	3	10
G72	2.225 ± 0.223 (1) a	3.593 ± 0.285 (1.61) ***	4.785 ± 0.249 (2.15) ***	8.056 ± 0.769 (3.62) ***
G72_123_**_–_**_153_	2.225 ± 0.223 (1)	2.360 ± 0.219 (1.06) *	2.563 ± 0.157 (1.15) **	2.710 ± 0.063 (1.22) ***
G72_138_**_–_**_153_	2.225 ± 0.223 (1)	2.425 ± 0.205 (1.10) **	2.430 ± 0.075 (1.10) **	2.938 ± 0.118 (1.32) ***
LL37	2.225 ± 0.223 (1)	2.280 ± 0.176 (1.02)	2.386 ± 0.037 (1.07)	2.271 ± 0.035 (1.02)
3× FLAG	2.225 ± 0.223 (1)	2.106 ± 0.042 (0.95)	2.305 ± 0.149 (1.04)	2.319 ± 0.236 (1.04)

a The numbers in the parentheses are the relative fold changes in DAO activity expressed as *V*_0_ in the presence of G72 or a truncated form of G72 divided by *V*_0_ in the absence of G72 or a truncated form of G72.

* *p* < 0.05;

** *p* < 0.01;

*** *p* < 0.001.

**Table 2.** Primers used in this study.

ID	Sequence
DAO-R′-*Xho*I	5′-TATA**CTCGAG**GAGGTGGGATGGTGGCATTC-3′
DAO-F′-*Nde*I	5′-ATAT**CATATG**CGTGTGGTGGTGATTGG-3′
G72-F′-*Nde*I	5′-ATAT**CATATG**CTGGAAAAGCTGATGGG-3′
G72-R′-*Xho*I	5′-TATA**CTCGAG**TTCAGCTTTGGTAGAAG-3′
G72-R′-*Pst*I	5′-TATA**CTGCAG**TTCAGCTTTGGTAGAAG-3′
G72_95–153_-F′-*Nde*I	5′-ATAT**CATATG**CTTGAAGAAGTAAGCAG-3′
G72_1–137_-R′-*Pst*I	5′-TATA**CTGCAG**TTGCTGGTTGTAGTTGC-3′
G72-60-F′	5′-TCCAGATATACATTG**TGATCA**AAAATCTACTTC-3′
G72-60-R′	5′-GAAGTAGATTTT**TGATCA**CAATGTATATCTGGA-3′
G72-105-F′	5′-AGCATTCTTCTGAGC**TGATCA**TCTGAAGGCTCT-3′
G72-105-R′	5′-AGAGTTTTCAGA**TGATCA**GCTCAGAAGAATGCT-3′
G72-180-F′	5′-ACAAGGAAAGAAGGA**TGATCA**AAGAGAAGGCAT-3′
G72-180-R′	5′-ATGCCTTCTCTT**TGATCA**TCCTTCTTTCCTTGT-3′
G72-270-F′	5′-TCTTACCTTCCTCAG**TGATCA**TATGCAGAGCTT-3′
G72-270-R′	5′-AAGCTCTGCATA**TGATCA**CTGAGGAAGGTAAGA-3′
G72-366-F′	5′-GCCTCTAAGGACCGC**TGATCA**CAGCCTCTAGAA-3′
G72-366-R′	5′-TTCTAGAGGCTG**TGATCA**GCGGTCCTTACAGGC-3′

The bolded characters are restriction enzyme sites. The sites underlined are *Bcl*I.
