# Supplementary material for: The C-Terminal Region of G72 Increases d-Amino Acid Oxidase Activity
Source: Int J Mol Sci. 2013 Dec 20;15(1):29–43. doi: 10.3390/ijms15010029 (PMC3907796; doi:10.3390/ijms15010029)
Supplement: Supplementary file 1 [file ijms-15-00029-s002.pdf]

Supplementary Information

Figure S1. Prediction of the G72 secondary structure. The secondary structure of G72 was predicted using Hierarchical Neural Network at the ExPASy proteomics server. h, e and c: Residues predicted to have an -helical, -strand, or coil conformation, respectively.

View HNN in: [AnTheProt (PC) , Download...] [HELP]

```
        10        20        30        40        50        60        70
         |         |         |         |         |         |         |
MLEKLMGADSLQLFRSRYTLGKIYFIGFQRSILLSKSENSLNSIAKETEEGRETVTRKEGWKRRHEDGYL
cchhhccchhhhhhhcccccceeeeeeechheeecccccchhhhhhccccccceeeecccccchchhhhhh
EMAQRHLQRSLCPWVSYLPQPYAELEEVSSHVGKVFMARNYEFLAYEASKDRRQPLERMWTCNYNQQKDQ
hhhhhhhhhhhhccccccccccchhhhhhhccchhhhhhhhhhhhhhhccccccchhhhhhhcccccccc
SCNHKEITSTKAE
ccccccccccccc

Sequence length :   153

HNN :
   Alpha helix      (Hh) :   66 is  43.14%
   3_10 helix       (Gg) :    0 is   0.00%
   Pi helix         (Ii) :    0 is   0.00%
   Beta bridge      (Bb) :    0 is   0.00%
   Extended strand  (Ee) :   14 is   9.15%
   Beta turn        (Tt) :    0 is   0.00%
   Bend region      (Ss) :    0 is   0.00%
   Random coil      (Cc) :   73 is  47.71%
   Ambigous states  (?)  :    0 is   0.00%
   Other states          :    0 is   0.00%
```

© 2013 by the authors; licensee MDPI, Basel, Switzerland. This article is an open access article distributed under the terms and conditions of the Creative Commons Attribution license (http://creativecommons.org/licenses/by/3.0/).
